# Immunophenotypic Profiles in Polycystic Ovary Syndrome

**DOI:** 10.1155/2020/5894768

**Published:** 2020-03-19

**Authors:** Cong Hu, Bo Pang, Zhanchuan Ma, Huanfa Yi

**Affiliations:** ^1^Central Laboratory of the Eastern Division, The First Hospital of Jilin University, Changchun, Jilin, China; ^2^Key Laboratory of Organ Regeneration and Transplantation, Ministry of Education, Changchun, Jilin 130021, China; ^3^Center for Reproductive Medicine, Center for Prenatal Diagnosis, The First Hospital of Jilin University, Changchun, Jilin, China; ^4^Department of Cardiology, The First Hospital of Jilin University, Changchun, Jilin, China

## Abstract

Polycystic ovary syndrome (PCOS) a long-known endocrinopathy and one of the most common endocrine-reproductive-metabolic disorders in women, which can lead to infertility. Although the precise etiology remains unclear, PCOS is considered as a complex genetic trait, with a high degree of heterogeneity. Besides, hormones and immune cells, including both innate and adaptive immune cells, are reportedly a cross talk in PCOS. Chronic low-grade inflammation increases autoimmune disease risk. This proinflammatory condition may, in turn, affect vital physiological processes that ultimately cause infertility, such as ovulation failure and embryo implantation. Here, we review the accumulating evidence linking PCOS with inflammatory status providing an overview of the underlying hormone-mediated dysregulation of immune cells. We mainly focus on the correlational evidence of associations between immune status in women and the increased prevalence of PCOS, along with the specific changes in immune responses. Further recognition and exploration of these interactions may help elucidate PCOS pathophysiology and highlight targets for its treatment and prevention.

## 1. Introduction

Polycystic ovary syndrome (PCOS) is one of the most common endocrine-reproductive-metabolic disorders in females since prehistory and remains a major cause of infertility, affecting approximately 5–15% of women worldwide [1]. The main clinical manifestations of PCOS are obesity and hyperinsulinemia/insulin resistance [2], irregular menstruation, and oligo-/anovulation. The primary hormonal abnormalities in PCOS are characterized by higher androgen and estrogen but lower progesterone levels [3]. Several factors have been associated with PCOS development, which might ultimately lead to female infertility because of failed follicles maturation and embryo implantation [4]. PCOS etiology is not clear and is considered a complex genetic trait, characterized by a high degree of heterogeneity [5]. Furthermore, PCOS can be associated with a series of complications. For example, the incidence of gestational diabetes, asthma, and recurrent miscarriage is 3–7-fold, 10-fold, and 3–5-fold higher in women with PCOS than in the general population, respectively [6, 7].

A recent study has shown that a higher body mass index is associated with hypertriglyceridemia [8] in PCOS patients, which is attributed to the obesity-induced change of adipokines, including tumor necrosis factor-alpha (TNF*α*), interleukin (IL)-6, and adiponectin [9]. The risk factors of PCOS are linked to a sedentary lifestyle and western-style eating habits, which can lead to fat accumulation, in turn, contributing to the recruitment of immune defense cells [10]. This situation has been described as a mild yet chronic proinflammatory condition in obese patients, affecting not only the adipose tissue but also other target organs, such as the ovary [11]. The number of peripheral white blood cells is also significantly increased in PCOS patients, as compared with that of healthy controls. In addition, the proportion of immune cell subgroups, such as lymphocytes, macrophages, and eosinophils, is significantly elevated in PCOS [12]. These alterations may be further exacerbated by the obesity condition that is present in a high percentage of PCOS patients. Besides, inflammatory reactions can influence vital physiological processes such as ovulation [13] and embryo implantation [14]. Even childhood obesity (before 12 years of age) seems to potentially increase the risk of female infertility later in life [15].

Furthermore, it has been proposed that PCOS may be associated with autoimmune diseases. A correlational analysis has shown that women account for seventy-eight percent of the population suffering from autoimmune diseases, and this may be associated with estrogen levels, as the onset of autoimmune disease occurs at an earlier age in women than in men [16]. Moreover, the interactions between the ovary, immune cells, and their products such as steroids, peptide hormones, prostaglandins, growth factors, and cytokines play pivotal roles in the regulation of ovarian function [17]. Here, we reviewed the accumulating literature on the relationship between PCOS and immune cells and their impacts on metabolic and reproductive disorders, which may provide a better understanding of PCOS etiology.

## 2. Innate Immune Cells and PCOS

### 2.1. Macrophages

Macrophages are the most abundant immune cells within the adipose tissue and ovary, both in animals and humans [18, 19], particularly in the thecal, luteal, and atretic follicles, where they participate in multiple processes in the ovary, such as folliculogenesis and ovulation [19, 20]. Macrophages are also crucial for maintaining a balance between protective and destructive cell-mediated immunity in the healing phase of inflammation [21]. Both endogenous and environmental factors seemingly affect macrophage populations in human peripheral tissues. Their distribution fluctuates throughout the ovarian cycle with the highest numbers observed at the ovulation and luteal phases, showing evidence of hormonal regulation [19]. A previous study has reported that macrophage loss was accompanied by induction of several proinflammatory genes, which is reminiscent of the physiological process of luteolysis, and the luteal phase progesterone deficiency is insufficient to provide trophic support for the formation of the vascular network, which is critical to corpus luteum function [21]. The dysfunction of fat cells and accumulation of macrophages can also result in an influx of a plethora of proinflammatory cytokines and chemokines (e.g., IL-1, IL-6, IL-10, IL-12, nitric oxide, and TNF*α*) into the circulatory system at the same time, leading to a state of systemic, chronic low-grade inflammation that can affect ovarian function [22].

Obesity and insulin resistance are clinical manifestations of PCOS, which is also characterized by a transition in macrophage polarization from an alternative anti-inflammatory M2 state to a proinflammatory M1 state. At the same time, M1 macrophages inhibit insulin sensitivity by producing inflammatory cytokines, such as TNF*α* and IL-6, whereas M2 macrophages exert the opposite effect [23, 24]. There are higher levels of TNF*α* and IL-6 both in the serum and particularly in the follicular fluid in PCOS [25], suggesting that the follicular granulosa cells may be involved in secreting these cytokines.

TNF*α* is not only a proinflammatory cytokine and participates in obesity-related systemic insulin resistance by inhibiting tyrosine kinase of the insulin receptor in muscle and fat but also known to be indispensable for follicular formation, oocyte maturation, and androgen synthesis and to mediate insulin resistance [26]. As it plays a crucial role in the apoptosis of the granulosa and luteal endothelial cells, finally leading to follicular atresia and a luteolytic effect, its concentration determines the quality of the oocyte [27] and eventually promotes PCOS-independent hyperandrogenemia and obesity [28]. When TNF*α* binds to its receptor (TNFR1) in macrophages, caspase-8 and caspase-3 are cleaved and activated, thereby inducing I*κ*B phosphorylation and its degradation to activate nuclear factor *κ*B (NF*κ*B). Subsequently, NF*κ*B translocates to the nucleus where it can activate the transcription of certain genes, particularly those involved in immune and inflammatory responses [29]. In addition, IL-6 was shown to attenuate estradiol production, partially by inhibiting the expression of aromatase in rat granulosa cells [30, 31]. Thus, it is plausible that increased IL-6 expression in PCOS may contribute to the steroidogenic ability to, in turn, decrease the androstenedione conversion to estradiol in the ovary. A comparison of cultured macrophages from rats showed that the levels of TNF*α* and IL-6 secretion increased in the testosterone-treated PCOS group but slightly declined in response to estrogen treatment, whereas progesterone treatment had no effect [32]. As TNF*α* and IL-6 also potentially induce insulin resistance, stimulate the production of androgen, and cause hypothalamic-pituitary-ovarian axis secretion disorder, a concomitant PCOS condition may result in a vicious cycle [33]. Therefore, we consider that prolonged high androgen levels experienced by PCOS patients might drive macrophages conversion to the M1 phenotype, resulting in the secretion of more proinflammatory cytokines and thereby enhancing PCOS clinical manifestations.

Macrophages also secrete migration inhibitor factor (MIF) [34, 35], which is the first proinflammatory cytokine discovered. MIF may inhibit insulin secretion by inhibiting the phosphorylation of tyrosine in the adipose tissue and the insulin receptor substrate in insulin signal transduction [36]. Matsuura et al. [37] demonstrated that anti-MIF antibody could inhibit follicle growth and ovulation in rats. Moreover, the MIF level in the circulation fluctuates during the menstrual cycle and positively correlated with the level of luteinizing hormone (LH), which can explain why MIF levels are higher in PCOS patients than in healthy controls [35]. However, Covington et al. postulated a different conclusion, demonstrating that *MIF* and *IL-6* mRNA levels in the adipose tissue of PCOS patients were lower than those of healthy controls, with no difference in *TNFα* levels between the groups [38]. These contradictory findings suggest that macrophages from various sources may release entirely different levels of cytokines.

### 2.2. Dendritic Cells (DCs)

DCs are a heterogeneous group of antigen-presenting cells, which exist in an immature state in the circulation and have potent phagocytotic ability; thus, they can capture and process antigens and present them to T cells in the lymph nodes, serving as a bridge between the innate and adaptive immune responses [39]. After receiving the activation signal associated with the antigen, DCs produce cytokines and inflammatory mediators such as TNF*α*, IL-6, IL-11, IL-12, and IL-23, which, in turn, induce the proliferation of allogeneic T cells and differentiate them to the Th17 and Th1 subtypes [40]. However, in visceral adipose tissue (VAT), DCs suppress inflammation by activating the *β*-catenin and PPAR*γ* pathways, which are important regulatory mechanisms for fat expansion [41]. Subsequently, *β*-catenin activation triggers PI3K/Akt that, in turn, induces IL-10 production and inhibits IL-6 secretion [42]. By contrast, accumulating evidence implicates CD11c^+^HLA-DR^+^ DCs as important cell components of the follicular fluid, and mature DCs were positively correlated to the ovary reaction to gonadotrophic, suggesting a function related to the aseptic inflammation in ovulation [43, 44]. Also, the number of DCs in the follicular fluid was found to be significantly decreased in PCOS patients as compared to those in healthy controls [45]. Therefore, we consider that with more VAT in PCOS patients, DCs may serve not only to restrain obesity-induced inflammation but also to promote pathogen persistence. Meanwhile, there might not be a sufficient amount of DCs in the follicular fluid to induce the recruitment and activation of T cells (Th17, Th1 cells), resulting in the failure of follicle development and maturation. Future studies to investigate these possibilities will be of extreme importance.

### 2.3. Innate Lymphoid Cells (ILCs)

ILCs develop from common lymphoid progenitor cells whose morphology resembles that of adaptive lymphocytes. Recently, ILCs are being recognized as critical modulators of tissue homeostasis and inflammation via cytokine release [46]. ILCs can be divided into three groups based on the expression of transcriptional factors and cytokines [47]: group 1 ILCs, which include natural killer (NK) cells and ILC1s; group 2 ILCs, which consist of ILC2; and group 3 ILCs, which consist of lymphoid tissue inducer cells and NKp46^−^ and NKp46^+^ ILC3s [48].

NK cells possess microbicidal activity against a diverse group of pathogens, which not only kill tumor cells and microbes but also regulate the activity of other immune cells, such as macrophages and DCs [49]. NK cells are barely detectable in both the intra-follicle and peri-follicle cells of PCOS patients and healthy controls [50]. CD3^−^/CD56^+^ granule lymphocytes are the uterine NK cells (uNK), which lack CD16 expression and have high expression of CD56 (CD56^bright^), unlike CD3^−^/CD56^+^/CD16^+^peripheral blood NK cells (PBNK) [51]. The normal uterine endometrium is decidualized by the effect of progesterone, which is associated with the homing and proliferation of PBNK, while the uNK cells do not express progesterone receptors [52]. Progesterone can also regulate the expression of CXCL10, IL-15, and IL-18 in the process of endometrium decidualization [53]. Moreover, androgen receptor suppresses *IL12a* expression at the transcriptional level via direct binding to the *IL12a* promoter region, thereby repressing the efficacy of NK cell cytotoxicity; after androgen receptor antagonist treatment, the IL12A signals are elevated, and NK cell function is enhanced [54]. With high androgen and reduced progesterone, PCOS patients have decreased CXCL10, IL-15, IL-18, and IL-12A levels, which play important roles in maternal-fetal tolerance and maintenance in pregnancy, suggesting that impairment in recruiting NK cells in PCOS patients may lead to a cytokine disorder. The receptivity of the endometrium is a precondition for a successful pregnancy. Thus, NK cells might explain infertility associated with PCOS, besides the main manifestations of follicular dysplasia and ovulation disorder.

Group 3 ILCs (ILC3) produce Th17- and Th22-like cytokines IL-22, IL-17, and ROR*γ*t [55]; in PCOS, it directly correlates with serum androgen concentrations and inversely with estradiol levels [56]. Flow cytometry shows a reduction of ILC3 (CD45^+^IL-22^+^ROR*γ*t^+^) in both intestinal and blood samples in PCOS individuals and PCOS-like animal models; furthermore, it also exhibits a therapeutic role in PCOS [57]. It is well-known that the gut microbiota and its metabolites may contribute to glucose homeostasis through immune system modulation [58]. Besides, a remarkable abundance of gram-negative anaerobic bacteria inhabits the distal human gut in individuals with PCOS and negative correlated with ILC3 proportion [59]. If transplant interspecific fecal from PCOS women to adult mice, female recipient mice would exhibit the major PCOS cardinal defects: hyperandrogenism, high LH secretion, impairment of reproductive cycles, ovarian dysfunction, and insulin resistance [57]. Though ILC3 functions as a double-edged sword in some autoimmune diseases [60], it can alleviate PCOS progression. However, the inability to precisely distinguish ILC3 and Th17 remains the main paradox in the field; if Th17 is elevated in PCOS, why ILC3 is reduced?

## 3. Adaptive Immune Cells in PCOS

The adaptive immune system comprises T cells and B cells. T cells are involved in cell-mediated immune responses, whereas B cells mainly mediate the humoral immune response. Since there are very few B cells in the female genital tract [61], only minimal research on the relationship between B cells and PCOS has been conducted. Therefore, in this section, we focus only on potential relationships and mechanisms concerning T cells and their subpopulations.

In normal circumstances, the selection of dominant follicles and the apoptosis of nondominant follicles is an essential mechanism to maintain homeostasis of the ovary [62]. Many factors are related to the survival of follicles during the follicle decrease stage. Specifically, T cells play a crucial role in mediating inflammation and insulin resistance by secreting proinflammatory cytokines in various metabolic organs and promoting follicles by releasing specific chemokines and growth factors to promote granular cell development and selection of the ovarian follicles, along with cytotoxic signals to induce the apoptosis of granulosa cells [63]. It has been found that the testosterone level and the number of CD45RO^+^ cells negatively correlate in the sinus follicle theca cells of PCOS patients and controls [64]. In particular, the PCOS group showed abnormally high androgen levels, as a characteristic of the condition, along with significantly decreased CD45RO^+^ T lymphocytes (activation/memory T lymphocytes). Another study [65] has shown that 5–7 days old mice were injected with estrogen, androgen, or progesterone; mice in the former two groups had decreased numbers of thymus cells 12 days later, whereas the thymus cell population increased in the progesterone-treated group. Moreover, the CD4^+^CD8^+^ double-positive T cells and CD4^+^ and CD8^+^ single-positive T cells of the adult mice were reduced by 99%. Therefore, inadequate distribution of T cells might lead to the failure of natural follicular selection and PCOS development.

### 3.1. T Helper (Th) Cells

CD4^+^ Th cells are central orchestrators of proinflammatory and anti-inflammatory immune responses. Activated CD4^+^ T cells are triggered to differentiate into Th cells, guided by specific costimulatory signals and the cytokine *milieu* [66]. IL-12 can drive the differentiation of T cells to Th1 cells, which is an immune-invasive subpopulation [67]. In contrast, IL-13 and IL-4 orchestrated actions drive the differentiation of T cells to Th2 cells, which mediate immune tolerance [68]. Circulating androgen and estradiol highly correlate with circulating inflammation [69], as IL-13 levels in the follicular fluid of PCOS patients were found to be significantly lower than those of women with regular ovulation, whereas the concentration of IL-12 increased significantly [70], which could induce a shift from Th2 to Th1 cells [71, 72]. Moreover, estrogen was shown to augment the secretion of inflammatory cytokines such as TNF*α*, IL-6, and interferon-gamma (IFN*γ*) in Th1 lymphocytes, whereas the progesterone spike in the luteal phase decreased these levels [72]. Due to the accumulation of numerous follicles with no ovulation, patients with PCOS show a high level of estrogen without progesterone resistance. IL-6 can also stimulate the expression of the key transcription factors of Th17 cells by activating STAT3 and the expression of ROR*α* and ROR*γ*, thereby promoting the differentiation of Th0 cells to Th17 cells [43, 73]. Though some information shows Th17 of PCOS is decreasing in animal models [74, 75], in human PCOS, evidence focuses on the expansion of proinflammatory Th17 subset not only in the blood but also kidneys [75]; however, the reason is obscure. In this condition, IL-6 may inhibit TNF*α* production and also effectively drive angiogenesis, thus promoting the formation of blood vessels and increases the concentration of the follicle-stimulating hormone [76]. It is confirmed that a significant difference in the Th17/Th2 ratio, with a bias toward Th17, is common among patients with PCOS [77]. Thus, the accumulation of Th1 and Th17 cells leads to immune overaction, which implies that PCOS might have an autoimmune origin.

### 3.2. Cytotoxic T (Tc) Cells

Tc cells are the primary effector cells of the cellular immune system. They induce cytotoxic processes to eliminate infected or malignantly transformed cells. These effects are brought by cytokine secretion, the release of cytotoxic agents and direct cell-cell contact [78]. It has been reported that changes in lymphocyte subgroups are associated with hormone levels [43]. In particular, increased androgen level could affect the endocrine and immune systems and resulted in a 64% decline of CD8^+^ T cell counts in PCOS patients [79].

CD4^+^CD28^null^ is a subgroup of cytotoxic T cells with proinflammatory function, producing high levels of IFN*γ*, TNF*α*, IL-2, and cellular enzymes, representing states of chronic inflammation and persistent infection, which may lead to the loss of CD28 on the cell surface [73]. These T cells cannot induce B lymphocyte activation and produce antibodies but have cytotoxic features [80]. Thus, CD4^+^CD28^null^ cells are rarely found in healthy individuals and are primarily associated with various inflammatory diseases [81]. Tc number is significantly increased in PCOS patients compared with those of controls [82]. However, recent studies show that CD4^+^CD28^null^ cells are not associated with hyperinsulinemia, high-sensitivity C-reactive protein (hsCRP) levels, obesity, and androgen levels of PCOS but only with the general PCOS status [25] and exhibit high proinflammatory and tissue-damaging properties [73]. All of these indicate that PCOS may be related to a general decline of the immune response.

### 3.3. Regulatory T Cells (Tregs)

Tregs can be divided into two groups: naturally occurring regulatory T cells (nTregs), produced by the thymus gland, and induced regulatory T cells (iTregs) that originate from the peripheral lymphoid tissues. In human peripheral blood, CD4^+^CD25^+^CD127^-/low^Foxp3^+^ Tregs account for approximately 1–2% of the total CD4^+^ T cells, helping to prevent autoimmune diseases by inhibiting the proliferation of effective T cells and cytokines production [83]. Tregs thus play an essential role in immune tolerance in healthy states, and their dysregulation is strongly associated with the development of autoimmune diseases.

As progesterone is a crucial regulatory factor for the development and production of peripheral Tregs [84], which negatively correlated with IL-6 level, it facilitates the generation of Foxp3 that subsequently affects Tregs production [84]. Tregs are decreased in the ovulation phase and are found at the highest level in the luteal phase [85]. The Tregs produced during ovulation and in the post-ovulation phase of the cycle are essential for the immune tolerance of the embryo after implantation. Indeed, a decrease of Tregs is related to the occurrence of spontaneous abortion [86], unexplained recurrent abortions [87], and preeclampsia [88]. In addition, the number of Tregs in the peripheral blood of PCOS patients was shown to be lower than that of controls [89]. Therefore, the Th17/Tregs ratio would increase, leading to a chronic inflammatory state in the ovary and throughout the body.

In mice, estrogen carries out its functions through estrogen receptors on CD4^+^CD25^−^ T cells, promoting their transformation to CD4^+^CD25^+^ T cells during the embryonic period to increase the quantity of Tregs [90]. By contrast, other researchers [83] have proposed that androgen can directly combine with AR through complementary sequence pairing to directly regulate target genes or indirectly, through its metabolites, to induce higher Foxp3 expression during ovulation.

**Figure 1:**
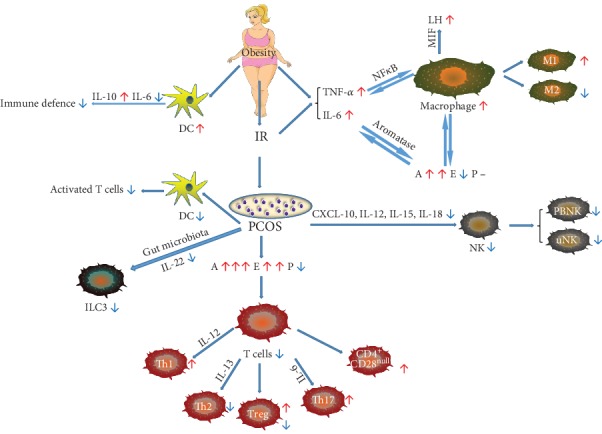
Relationship between PCOS and immune cells. (1) PCOS is often associated with obesity and IR. (2) Obesity and IR elevate proinflammatory cytokine release, such as TNF*α* and IL-6, which lead to an increase in macrophages through the NF*κ*B pathway, resulted in A upregulation, while E downregulation, and with no interference of P, via inhibiting aromatase activation. (3) PCOS status can decrease NK cells, both PBNK and uNK, through downregulation of CXCL-10, IL-12, IL-15, and IL-18 cytokines. (4) Obesity causes an increase in DCs, and PCOS status decreases DC number, both leading to the destruction of immune defenses. (5) The steroid hormone alterations of PCOS lead to a decrease in T cells and changes in subgroup proportions. IR: insulin resistance; PCOS: polycystic ovarian syndrome; TNF*α*: tumor necrosis factor-*α*; IL: interleukin; NF*κ*B: nuclear factor *κ*B pathway; MIF: migration inhibitor factor; DCs: dendritic cells; PBNK: peripheral blood NK cells; uNK: uterus NK cells; ILCs: innate lymphoid cells; Th: helper T cells; Treg: regulatory T cells; A: androgen; E: estrogen; P: progesterone.

In summary, hyperestrogenism, hyperandrogenism, and hypoprogesterone play an essential role in the dynamic change of Tregs in PCOS. Since this would increase the Tregs counts in females, this mechanism could offer a new therapeutic strategy for autoimmune diseases (Figure 1).

## 4. Other Immunological Mechanisms of PCOS

The white blood cell counts in the peripheral blood of patients with PCOS and hyperinsulinemia increased along with hyperandrogen production [91] and mainly increased in macrophages and neutrophils, which infiltrate this excessive fat to “clean up” dysfunctional and dead cells, resulting in a state of chronic low-grade inflammation [11]. As PCOS is usually treated with oral contraceptive and metformin [92], the number of macrophages and neutrophils may become even higher under treatment with oral contraceptive monotherapy and could be improved with metformin [91]. Besides its better-known effects in the improvement of oxidative stress and insulin resistance, metformin is also an effective treatment for immune-related disorders. However, the ability of metformin to improve the clinical signs and symptoms of PCOS by immunological mechanisms requires further research.

Though B cells in PCOS patients are poorly detected, there are significant differences in antinuclear antibody, resistance to histone antibody, and ds-DNA antibody levels in 109 PCOS patients and controls [93]. Furthermore, patients with PCOS have a higher incidence of autoimmune thyroiditis, which was associated with increased thyroperoxidase or thyroglobulin antibody levels [94]. These further observations demonstrate that due to the immune microenvironment imbalance, PCOS can coexist with or even cause other autoimmune diseases.

In addition, the role of minerals like calcium and vitamin D in the development of many diseases has been evaluated, especially endocrine, inflammation, and oxidative stress, recently [95, 96]. A plethora of Ca^2+^-permeable channels in T cells at various locations, with unique activation mechanisms, have been reported to be necessary for T cell activation, maturation, and secretion of cytokines [97]. Not only Ca^2+^ can activate human monocytes to produce inflammatory cytokines and promote M1 macrophage development [98], but Ca^2+^ influx is also vital in the proinflammatory functions of neutrophils, which promotes autoimmune and inflammatory disease progression and exacerbates collateral damages to the host tissues [99]. The change and function of Ca^2+^ in PCOS still need further research. Furthermore, vitamin D's anti-inflammatory impact on human pathophysiology is well-accepted [100]. A current meta-analysis of randomized controlled trials concluded that vitamin D supplementation to women with PCOS results in an improvement in hsCRP, malondialdehyde, and total antioxidant capacity [95, 96]; serum total testosterone and androstenedione levels were reportedly lowered in vitamin D-calcium cosupplement group as compared to the control group [101], in response to upregulated insulin receptor genes [102]. However, vitamin D does not affect the symptoms of hyperandrogenism [103]. Additional studies should address the function of vitamin D on different subgroups of immune cells and illustrate the exact underlying mechanism.

Due to the abnormal hormone and irregular ovulation, miscarriage rate of patients with PCOS is higher than that of healthy subjects [104]. In addition to the cells mentioned above, other recently identified immune cells, such as T follicular helper cells (Tfh), Th9, Th22, and myeloid-derived suppressor cells (MDSCs), also might be involved in PCOS. Tfh are increased in both recurrent spontaneous abortion [105] and preeclampsia patients [106] as compared with healthy pregnant women, whereas MDSCs decreased [107, 108]; however, the interplay and relative changes that ultimately redirect and conduct PCOS are still unclear. Further research is warranted to elucidate the relationship between PCOS and immune cells.

## 5. Conclusions and Prospects

In this review, we highlight recent studies demonstrating a likely link between immune dysregulation, hormones, and PCOS. In particular, obesity with a higher level of estrogen and androgen may cause persistent immune system stimulation in PCOS patients, leading to proinflammation cell increase, such as M1, Th1, and Th17; and anti-inflammation cells decrease, such as M2 and Tregs; meanwhile, the antigen-presenting cells change dichotomic. Immune microenvironment imbalance results in the production of autoantibodies to trigger autoimmune diseases. It is also possible that the immune tolerance breakdown causes the body to rest in a chronic inflammatory state, which affects the generation, development, and follicular ovulation. In recent years, the clinical symptoms of PCOS have been mainly treated by exercise, along with an oral insulin sensitizer and anti-inflammatory molecules, such as metformin, and oral contraceptives, which have all improved the short-term prognosis of ovulation. However, more in-depth research should be conducted to understand the precise etiology of PCOS and to develop more effective and targeted treatments. Recent evidence summarized in this review points to the potential of improving the basic immune state to enhance PCOS treatment. Thus, research efforts on PCOS about the underlying immunological mechanisms could help us discover a novel targeted treatment in the near future. In the next decade, several issues regarding the immune treatment, outlined in this study, shall be addressed to explore beyond the experimental framework summarized herein and to provide novel therapeutic targets for clinical practice.
